# Ablation in Complex Adult Congenital Heart Disease: Versatility Matters

**DOI:** 10.1016/j.jaccas.2026.107767

**Published:** 2026-04-15

**Authors:** Laurent Roten

**Affiliations:** Department of Cardiology, Inselspital, Bern University Hospital, University of Bern, Bern, Switzerland

**Keywords:** ablation, congenital heart defect, supraventricular arrhythmias

Catheter ablation of atrial tachyarrhythmias in adults with congenital heart disease (ACHD) remains among the most technically demanding procedures in electrophysiology. Several factors converge to make these cases uniquely challenging: venous access may itself be the first hurdle; anatomy is difficult, unusual, and highly individual; access to target chambers can be complex and restricted, particularly in Fontan and atrial-switch patients; and general anesthesia is frequently necessary because tachycardia, limited preload reserve, and respiratory compromise can rapidly destabilize patients.^1^^,^^2^ This results in demanding and often lengthy procedures that require deep operator expertise, careful planning, and a low threshold for adjunctive imaging.

Within this context, Pipilas et al^3^ in this issue of *JACC: Case Reports* elegantly illustrate how catheter versatility and procedural efficiency can become key determinants of feasibility and safety in complex ACHD ablation. Their patient, a 45-year-old man with double-inlet single ventricle post modified fenestrated Fontan, experienced recurrent intra-atrial re-entry tachycardia (IART) despite medical therapy and a prior ablation attempt using a conventional radiofrequency catheter. At redo, they again achieved transbaffle access and used the Affera/Sphere-9 platform to acquire sinus rhythm voltage and activation maps and then delineate the tachycardia circuit. Pulsed-field ablation (PFA) delivered from the systemic atrial aspect substantially slowed the tachycardia, but only iterative remapping within the baffle revealed earlier remnant atrial tissue adjacent to prior lesions; targeted PFA at this site terminated the tachycardia, which was noninducible thereafter, with arrhythmia-free follow-up reported at 10 months.

Their report verifies that these patients demand a mechanism-first mapping strategy. IART in ACHD is rarely “textbook” flutter; circuits are shaped by surgical scars, patch material, baffles/conduits, and hypertrophied tissue planes, often with multiple potential channels and bystander activation. A critical corollary is that mapping in sinus rhythm is not optional. Sinus activation mapping helps define how atrial activation reaches the atrioventricular node and reduces the risk of iatrogenic conduction impairment by ablating a corridor that is essential for sinus conduction in an already surgically constrained atrium.

Against this backdrop, the Sphere-9 catheter and its associated platform are particularly attractive. Even in its first iteration, the catheter allows for rapid generation of new voltage and activation maps and supports an iterative workflow (map, ablate, remap, refine) without repeated catheter exchanges, a critical advantage in ACHD anatomy, where each exchange may carry incremental risk. Mapping with this catheter is generally faster than point-by-point mapping using conventional ablation catheters, albeit slower than using multispline catheters; however, in ACHD, the ability to map and ablate with the same catheter, and to remap immediately after ablation, clearly outweighs raw acquisition speed.^4^

Energy flexibility adds another clinically meaningful dimension. The ability to toggle between radiofrequency ablation (RFA) and PFA allows tailoring of lesion creation to anatomy and collateral risk: RFA remains useful when predictable thermal lesion formation is desired and vulnerable structures are remote, whereas PFA may be preferred when targets lie near the phrenic nerve or esophagus or when a wider safety margin is prioritized.^5^ A dual-energy, map-and-ablate workflow is particularly compelling in ACHD, where arrhythmogenic tissue frequently sits adjacent to critical structures and procedural adaptation is routinely required.

Beyond atrial arrhythmias, such technologies are already expanding into other challenging ACHD substrates. We and others have used the same catheter for ventricular tachycardia ablation in ACHD populations and for ablation near mechanical valves, which may also be present in selected ACHD patients.^6^ Importantly, other PFA systems have also been used successfully to treat complex ACHD patients.^7^, ^8^, ^9^ In addition, other dual-energy map-and-ablate platforms are available and may prove particularly useful in complex ACHD ablation.^10^ However, the Affera platform, as well as other PFA systems, remain relatively new and lack a long track record in ACHD. Consequently, long-term outcome data are limited, and most current evidence derives from single-center experience or isolated case reports. In addition, these systems are associated with higher costs, which may limit broader adoption until clearer incremental clinical benefit is demonstrated.

Technological advances clearly benefit patients by improving procedural safety, efficiency, and ultimately effectiveness. The case presented by Pipilas and colleagues^3^ exemplifies how a versatile, relatively atraumatic, map-and-ablate catheter with dual-energy capability can simplify complex ACHD workflows while preserving a disciplined, mechanism-driven ablation approach. We have likely only begun this journey. As mapping fidelity improves and lesion technologies evolve, the ceiling for success in complex ACHD, particularly for deeper substrates such as ventricular tachycardia, should rise further, provided these tools are integrated thoughtfully with rigorous mechanism-based ablation strategy and careful attention to anatomic and physiologic constraints.

## Funding Support and Author Disclosures

Dr Roten has received research grants from Medtronic, the Swiss National Foundation, the Swiss Heart Foundation, the Immanuel and Ilse Straub Foundation, and the Sitem Insel Support Fund; and speaker fees/honoraria from Biosense Webster, Boston Scientific, Abbott, and Medtronic.
